# A Comparative Evaluation of Three Large Language Models for Parent‐Centered Questions About Anorexia Nervosa

**DOI:** 10.1002/eat.70118

**Published:** 2026-04-20

**Authors:** Celal Yeşilkaya, Hande Kırışman Keleş, Esra Rabia Taşpolat, Caner Mutlu, Serkan Turan

**Affiliations:** ^1^ Department of Child and Adolescent Psychiatry Konya Ereğli State Hospital Konya Türkiye; ^2^ Department of Child and Adolescent Psychiatry, Başakşehir Çam and Sakura City Hospital Health Sciences University Istanbul Türkiye; ^3^ Department of Child and Adolescent Psychiatry Iğdır Dr. Nevruz Erez State Hospital Iğdır Türkiye; ^4^ Department of Child and Adolescent Psychiatry, Faculty of Medicine Bursa Uludağ University Bursa Türkiye

**Keywords:** anorexia nervosa, artificial intelligence, child and adolescent mental health, digital health, health information, large language models

## Abstract

**Background:**

Large language models (LLMs) are increasingly used to obtain health information, including guidance on child and adolescent mental health. In anorexia nervosa (AN), where early recognition and timely intervention are critical, the accuracy of AI‐generated information available to parents may have important clinical implications. This study evaluated the performance of LLMs in responding to parent‐oriented questions about AN.

**Methods:**

A comparative model evaluation was conducted using three conversational AI systems: ChatGPT (GPT‐4o), Google Gemini, and DeepSeek. Twenty questions representative of those frequently asked by parents of adolescents with AN were identified through online content exploration and expert review. Each question was submitted using standardized prompts in separate chat sessions. Responses were anonymized and independently evaluated by two board‐certified child and adolescent psychiatrists across three dimensions: quality, usefulness, and reliability. Reproducibility was assessed through repeated queries in separate sessions.

**Results:**

All three models demonstrated generally high levels of reliability and overall informational performance. ChatGPT achieved the highest overall accuracy (≈92%) and reproducibility (≈90%), followed by Gemini (≈88% accuracy; ≈85% reproducibility) and DeepSeek (≈86% accuracy; ≈83% reproducibility). Domain‐level analysis showed lower accuracy across models for diagnosis and clinical assessment questions. Qualitative error analysis indicated that the omission of clinically relevant information was the most common limitation, while DeepSeek produced more factual inaccuracies and Gemini more generalized guidance.

**Conclusions:**

LLMs may provide broadly accurate preliminary information for parents seeking guidance about AN. However, persistent omissions and domain‐specific variability highlight important limitations. AI‐generated information should therefore be regarded as a complementary resource rather than a replacement for professional clinical guidance.

## Summary

- Many parents use online tools, including artificial intelligence chatbots, to look for information about eating disorders before consulting a healthcare professional.
- This study examined how accurately three widely used artificial intelligence systems—ChatGPT, Gemini, and DeepSeek—answered common parent questions about anorexia nervosa.
- Although the responses were generally accurate, important clinical information was sometimes missing or simplified.
- These findings suggest that artificial intelligence tools may help parents obtain preliminary information, but professional clinical guidance remains essential.

## 1 Introduction

The rapid development of artificial intelligence (AI) technologies, particularly large language models (LLMs), has created new opportunities for health information dissemination and patient or caregiver education. LLMs such as ChatGPT, Google Gemini, and DeepSeek have become widely accessible to the public, enabling individuals to seek medical guidance outside of traditional clinical settings. Beyond information generation, AI‐based approaches including machine learning algorithms have also shown promise in supporting diagnostic classification in child and adolescent psychiatry, such as differentiating patients with major depressive disorder from healthy controls based on neurocognitive profiles (Saglam et al. 2026). This change has significant implications for mental health, where timely access to accurate information can be critical for early intervention and family support.

Anorexia nervosa (AN) is a severe psychiatric disorder of childhood and adolescence associated with substantial psychiatric and medical morbidity. Lifetime prevalence has been estimated at up to 3.6% in women and 0.3% in men, and only about two‐thirds of affected individuals achieve long‐term recovery (Hebebrand et al. 2024). Recent evidence also indicates a marked increase in inpatient treatment rates for AN during the COVID‐19 pandemic, highlighting the disorder's sensitivity to environmental factors and the importance of timely recognition at the family level (Hebebrand et al. 2024). Early‐onset presentations introduce additional diagnostic challenges, as younger children may exhibit distinct clinical characteristics and evidence guiding age‐specific assessment and treatment for this population remains limited (Ayrolles et al. 2024). Even diagnostic thresholds, such as the ICD‐11 recommendation that AN‐related underweight be considered in relation to the fifth body mass index percentile for age, may be difficult for parents without clinical training to interpret. As a result, parents often occupy a critical but demanding role as first‐line observers of early warning signs, while their recognition of illness may be hindered by limited mental health literacy, stigma, and restricted access to specialized services.

Parents frequently use online resources when trying to understand their children's health problems. A systematic review evaluating 33 studies indicated that parents worldwide are heavy consumers of online health information for their children across highly diverse circumstances (Kubb and Foran 2020). Importantly, many parents did not routinely discuss the information they found with physicians and expressed a need for more guidance in identifying trustworthy resources (Kubb and Foran 2020). This pattern is particularly relevant in the field of child and adolescent mental health. A qualitative study involving parents of children with ADHD reported that online searches and social media communities played a central role in shaping parents' understanding of the diagnosis, navigating the care pathway, and managing differing professional perspectives (Bringer et al. 2025). Together, these findings establish that parents do not passively consume online health information; rather, they actively integrate it into their help‐seeking decisions in ways that can either facilitate or delay access to appropriate care. In conditions such as AN, where delayed recognition may lead to prolonged illness duration and serious medical complications, the quality of information obtained from digital sources may have important clinical implications.

Against this background, conversational AI tools represent an increasingly prominent source of health information. Emerging studies suggest that LLMs can provide responses that are often clear, structured, and broadly informative for parenting‐ and child health‐related questions, yet their performance remains inconsistent. Prior evaluations have identified important limitations, including inaccuracies or omissions in specific responses, limited citation transparency, insufficient developmental contextualization, and readability levels that may reduce accessibility for caregivers (Gugnani et al. 2024; Kim et al. 2025). A further concern involves citation fabrication: recent evidence indicates that GPT‐4o fabricates nearly one in five references in mental health literature reviews, with substantially higher rates for disorders that are less represented in the scientific literature (Linardon et al. 2025). Medical hallucinations more broadly—defined as outputs that are factually incorrect or unsupported by authoritative evidence—remain common across foundation models and are considered potentially harmful by a large majority of clinicians (Kim et al. 2025). These findings reinforce the importance of independent expert evaluation of LLM‐generated health information, rather than reliance on model outputs alone. Taken together, these studies suggest that while LLMs can approximate acceptable information quality for general parenting queries, their performance is uneven, and expert validation remains essential before these tools can be considered reliable guides in clinical contexts. A broader literature on pediatric mental health also suggests cautious optimism. In some structured assessments, leading LLMs have demonstrated relatively strong performance in psychiatric knowledge and mental disorder classification tasks (Hanafi et al. 2024; Neubauer et al. 2025). Other studies examining parent‐oriented questions in neurodevelopmental disorders similarly found that model‐generated answers may be useful but still require expert validation because of variability in quality and completeness (Almulla and Khasawneh 2025; Demir et al. 2025). These findings suggest that LLMs may approximate acceptable informational quality in some pediatric mental health contexts, but they cannot yet be assumed to provide consistently reliable guidance in clinically sensitive situations. Safety and communicative adequacy represent additional dimensions particularly salient in eating disorder contexts. The potential risks of inaccurate or poorly contextualized AI responses warrant serious concern, as parents may rely on these models to decide when to seek professional help or how to manage symptoms at home. Overreliance on such information can lead to dangerous delays in clinical assessment, the normalization of early warning signs, or inappropriate home management without expert guidance. In AN, where early intervention is vital and medical stability can decline rapidly, even minor inaccuracies, such as understating the need for medical monitoring or overlooking comorbid psychiatric symptoms, carry significant clinical risks. Furthermore, if a response lacks sufficient caution or fails to explicitly recommend professional evaluation, it may inadvertently reinforce parental hesitation. This could delay treatment and lead to poorer long‐term outcomes. Consequently, it is essential to evaluate LLM‐generated responses not only for their accuracy but also for their safety within high‐risk clinical contexts. Research evaluating LLMs on child and adolescent mental health myths found that while models correctly identified misinformation, responses were consistently rated as difficult to read below college level (Prazeres 2025).

Despite this growing literature, no study to date has specifically examined the performance of LLMs in response to parent‐initiated questions about AN in children and adolescents. This is an important gap. AN is diagnostically complex, early signs may be subtle, and misinformation may delay appropriate assessment or minimize the seriousness of symptoms. Given the increasing tendency of parents to consult digital resources before clinical contact, understanding how LLMs respond in this context is clinically relevant. The present study therefore evaluated the responses of ChatGPT, Google Gemini, and DeepSeek to 20 questions representative of those commonly asked by parents of adolescents with AN. In this study, informational accuracy was operationalized primarily through expert ratings of reliability, whereas quality and usefulness were examined as complementary dimensions reflecting educational value and practical helpfulness for parents. The primary aim was to assess the accuracy and reproducibility of information generated by these models. By identifying both strengths and limitations in model responses, this study seeks to inform clinicians, health communicators, and AI developers about the current utility of LLMs in a high‐risk pediatric mental health context.

## 2 Methods

### 2.1 Study Design

This study employed a comparative model evaluation design to evaluate the performance of LLMs in responding to questions commonly asked by parents of adolescents with AN. Three widely used conversational AI systems, ChatGPT (GPT‐4o), Google Gemini 3.0, and DeepSeek 3.2, were systematically evaluated. These three models were selected on the basis of their broad public accessibility, high global usage rates, and geographic diversity of origin, as well as their consistent use in comparable evaluations within the pediatric mental health literature. The study aimed to compare the informational quality, usefulness, and reliability of responses generated by these models in relation to clinically relevant questions about AN.

Because the study involved only the evaluation of AI‐generated responses and did not include human participants, patient data, or identifiable information, ethical approval was not required.

### 2.2 Question Identification and Selection

A semi‐structured search strategy was used to identify commonly asked parental questions about AN in adolescents. Questions were identified through a multi‐platform content exploration approach. Searches were conducted between January 2026–March 2026 using combinations of terms reflecting naturalistic parent concerns, such as “anorexia in teenagers,” “signs of anorexia in my child,” “is my child anorexic,” and “when to worry about eating habits.” For search engine queries, the first 2–3 pages of results were screened, as these are most likely to be accessed by lay users. For search engine queries, the first 2–3 pages of results were screened, as these are most likely to be accessed by lay users. Google Trends data were consulted to identify commonly searched AN‐related terms, which also helped guide the identification of relevant content (Coker et al. 2021). Additional sources included publicly accessible mental health websites and parenting resources, such as the National Eating Disorders Association (National Eating Disorders Association 2025) and Beat Eating Disorders (Beat Eating Disorders 2024), from which further question themes were derived. Platform selection was guided by the aim of capturing authentic parental information‐seeking behavior across diverse digital environments. To ensure clinical comprehensiveness, question themes were additionally informed by Fairburn's Cognitive Behavior Therapy and Eating Disorders (Fairburn 2008). Candidate questions were included if they (i) reflected a parental perspective, (ii) were relevant to adolescents with suspected or diagnosed AN, and (iii) addressed clinically meaningful aspects such as symptoms, diagnosis, treatment, or prognosis. Duplicate or highly similar questions were consolidated. The identified search terms and user‐generated content were used to inform the development of structured questions, which were subsequently refined for clarity and clinical relevance. The identification of commonly asked questions was guided by thematic recurrence across sources rather than a predefined quantitative frequency threshold. These sources were reviewed to capture frequently expressed parental concerns related to early warning signs, diagnostic procedures, treatment approaches, and long‐term outcomes.

Following the initial search process, candidate questions were independently reviewed by two clinicians specializing in child and adolescent psychiatry. Questions were selected based on their clinical relevance, frequency of appearance in public discussions, and coverage of key aspects of AN. A final set of twenty questions was compiled. The selected questions are presented in Table 1.

**TABLE 1:** Parent‐centered questions about anorexia nervosa.

Domain	Question
Early recognition and symptoms	
Q1	What are the earliest behavioral and physical warning signs that may indicate the development of anorexia nervosa in adolescents?
Q2	How can parents differentiate between normal dieting behaviors and clinically concerning restrictive eating patterns?
Q3	My child has recently started skipping meals and exercising excessively. Could these behaviors indicate anorexia nervosa?
Q4	What psychological changes, such as body image concerns or fear of weight gain, commonly appear before significant weight loss occurs?
Q5	Are there particular personality traits or risk factors that increase the likelihood of developing anorexia nervosa?
Diagnosis and clinical assessment	
Q6	At what point should parents seek professional evaluation if they suspect anorexia nervosa in their child?
Q7	What diagnostic criteria are used by clinicians to diagnose anorexia nervosa in adolescents?
Q8	Which medical and psychological assessments are typically performed during the diagnostic process?
Q9	How do healthcare professionals distinguish anorexia nervosa from other eating disorders or medical conditions causing weight loss?
Q10	What physical health complications may appear early during the course of anorexia nervosa?
Treatment and clinical management	
Q11	What evidence‐based treatments are recommended for adolescents diagnosed with anorexia nervosa?
Q12	How effective is family‐based treatment (FBT) in the management of adolescent anorexia nervosa?
Q13	When is hospitalization or inpatient treatment necessary for adolescents with anorexia nervosa?
Q14	Are medications useful in treating anorexia nervosa or its associated symptoms such as anxiety or depression?
Q15	How long does treatment for anorexia nervosa typically last, and what factors influence recovery outcomes?
Long‐term outcomes and family support	
Q16	What are the potential long‐term medical and psychological consequences of untreated anorexia nervosa?
Q17	Can adolescents fully recover from anorexia nervosa, and what does long‐term recovery usually involve?
Q18	How can parents support healthy eating behaviors and recovery at home during treatment?
Q19	What strategies can schools implement to support students recovering from anorexia nervosa?
Q20	How can parents cope with the emotional stress of supporting a child with anorexia nervosa?

*Note:* Q1–Q20 represent the parent‐centered questions used in this study to evaluate model responses. Questions were grouped into four domains: early recognition and symptoms, diagnosis and clinical assessment, treatment and clinical management, and long‐term outcomes and family support.

To enhance consistency in selection, the reviewers considered three guiding criteria: (i) clinical significance (potential to inform recognition or management of AN), (ii) recurrence across multiple sources, and (iii) coverage of core domains of the disorder. These criteria were applied qualitatively rather than as a formal scoring system.

The questions were categorized into four conceptual domains: (i) early recognition and symptoms, (ii) diagnosis and clinical assessment, (iii) treatment and clinical management, and (iv) long‐term outcomes and family support.

### 2.3 Model Prompt Control

To ensure methodological consistency and minimize prompt‐related bias, each question was submitted to the evaluated models using a standardized prompt structure. Each query was entered in a new chat session with no previous conversation history. All models were used with their default settings as provided in their respective user interfaces at the time of data collection, and no manual adjustments (e.g., temperature or response length) were applied. No follow‐up prompts, clarifications, or refinements were provided after the initial query. Each question was entered exactly as written, without additional instructions, role assignment, or contextual framing. In order to preserve ecological validity and to approximate real‐world user interactions with LLMs, the wording of the questions was intentionally designed to reflect how parents might naturally seek information about their child's condition.

All questions were asked in English using identical wording across models. The order of model querying was randomized to minimize potential temporal effects related to system updates or response variability.

### 2.4 AI Response Collection

Each of the 20 questions was submitted separately to the three evaluated LLM systems: ChatGPT, Google Gemini, and DeepSeek. The generated responses were recorded verbatim immediately after generation. No modifications or editing of the responses were performed prior to evaluation.

The complete response dataset was compiled and anonymized prior to expert review to prevent identification of the originating model.

### 2.5 Blinding Procedure

To reduce evaluation bias, responses were fully anonymized before scoring. Each response was assigned a randomized identification code and labeled only as Model A, Model B, or Model C. The evaluators were blinded to the identity of the generating model during the assessment process.

The order of responses was also randomized so that evaluators could not infer the model source based on response patterns.

### 2.6 Response Evaluation

The responses were independently evaluated by two board‐certified child and adolescent psychiatrists, each with more than 10 years of clinical experience in the assessment and management of eating disorders.

Three evaluation dimensions were assessed:

#### 2.6.1 Quality

Quality referred to the overall educational value of the response, including clarity, completeness, and the degree to which the explanation reflected clinically meaningful information relevant to parents and caregivers.

#### 2.6.2 Usefulness

Usefulness reflected how understandable and practically helpful the response would be for parents seeking guidance about AN.

#### 2.6.3 Reliability

Reliability assessed the factual accuracy and clinical consistency of the information provided, based on current evidence‐based knowledge and clinical guidelines for eating disorders.

An overall accuracy metric was computed as a composite score derived from quality, usefulness, and reliability ratings. Dimension scores were normalized to a common scale, averaged for each response, and then aggregated across questions. Final values were expressed as percentages to facilitate interpretation.

The definitions of these dimensions were informed by current evidence‐based clinical guidelines, specifically the National Institute for Health and Care Excellence (NICE) guidelines for eating disorders and the American Psychiatric Association (APA) Practice Guidelines, as well as relevant peer‐reviewed literature and the clinical consensus of the evaluating psychiatrists.

Quality was rated on a five‐point scale, while usefulness and reliability were evaluated using seven‐point Likert scales. On the five‐point quality scale, a score of 1 indicated a response with poor educational value, unclear structure, and major omissions, while a score of 5 indicated a comprehensive, well‐structured response with high educational value for parents. On the seven‐point Likert scales, a score of 1 indicated a response that was not at all useful (or not at all reliable), and a score of 7 indicated an extremely useful (or highly reliable) response fully consistent with current evidence‐based guidelines.

All responses were scored independently by both evaluators. Effect size estimates based on Cohen's d indicated small to moderate differences between models (quality: *d* = 0.68; reliability: *d* = 0.20–0.51; usefulness: *d* = 0.06–0.45).

### 2.7 Inter‐Rater Reliability

To assess agreement between the two evaluators, inter‐rater reliability was calculated using the intraclass correlation coefficient (ICC) with a two‐way random effects model for absolute agreement. ICC values were interpreted according to guidelines, where values below 0.50 indicated poor agreement, 0.50–0.75 moderate agreement, 0.75–0.90 good agreement, and values above 0.90 excellent agreement (Koo and Li 2016).

Disagreements between raters were resolved through discussion and consensus prior to final data analysis.

### 2.8 Data Reproducibility

To examine response stability, a subset of questions was repeated in separate chat sessions conducted on different days. Specifically, 6 out of the 20 questions were randomly selected and repeated. These questions were chosen to represent all four conceptual domains (early recognition, diagnosis, treatment, and long‐term outcomes), ensuring coverage of different types of clinically relevant content. This procedure allowed evaluation of the consistency of core informational content across repeated prompts. Core informational content was operationalized as the presence of consistent key clinical elements across responses, including: (i) similar diagnostic or clinical recommendations, (ii) comparable treatment or management guidance, and (iii) no major contradictions in the overall clinical message. Minor variations in wording, phrasing, or level of detail were not considered indicative of inconsistency. Responses were considered reproducible when the central clinical recommendations and key informational elements remained consistent across iterations.

### 2.9 Statistical Analysis

Descriptive statistics were calculated to summarize the performance of each model across the three evaluation dimensions. Mean scores and standard deviations were calculated for quality, usefulness, and reliability.

Item‐level descriptive statistics for each question are presented in Table 2. Comparisons between models were conducted using one‐way analysis of variance (ANOVA) to examine differences in mean scores across the evaluated dimensions. Effect sizes were calculated using Cohen's *d*.

**TABLE 2:** Descriptive statistics of quality, usefulness, and reliability indexes of answers generated for questions about anorexia nervosa.

Question	Quality			Usefulness			Reliability		
	ChatGPT	Gemini	DeepSeek	ChatGPT	Gemini	DeepSeek	ChatGPT	Gemini	DeepSeek
Q1	5	5	5	7	7	7	7	7	7
Q2	5	5	5	7	6	5	7	7	7
Q3	5	5	5	7	7	7	7	7	5
Q4	5	4	4	6	6	7	7	7	7
Q5	5	5	4	7	6	5	6	7	5
Q6	5	4	4	7	7	5	6	7	7
Q7	5	4	5	7	7	7	6	7	5
Q8	4	5	5	7	7	6	7	7	5
Q9	5	5	4	5	7	5	6	6	6
Q10	5	4	4	6	6	6	6	6	5
Q11	5	5	5	5	5	6	6	6	7
Q12	5	4	4	6	5	6	5	5	5
Q13	4	5	5	7	5	5	6	5	7
Q14	5	4	5	5	6	6	6	6	7
Q15	5	5	4	6	5	7	6	5	6
Q16	4	5	4	7	7	5	5	7	6
Q17	5	4	5	7	6	6	5	7	5
Q18	5	4	5	6	5	7	7	7	7
Q19	5	5	5	7	7	7	7	5	5
Q20	5	4	4	6	5	6	7	7	5

*Note:* Q1–Q20 represent the individual parent‐oriented questions about anorexia nervosa evaluated in this study. The full list of questions is presented in Table 1. Quality was rated on a 5‐point scale, while usefulness and reliability were rated on 7‐point Likert scales. Higher scores indicate better performance.

For reporting purposes, an accuracy rate was calculated for each model as the proportion of responses rated at or above a predefined threshold on the reliability scale (≥ 5 out of 7), reflecting responses judged to be clinically consistent and factually adequate. Similarly, reproducibility rates were calculated as the proportion of repeated queries in which core clinical content remained substantively consistent across sessions. These threshold‐based proportions were used to facilitate descriptive comparison across models and are reported alongside mean scale scores throughout the Results section.

All statistical analyses were performed using Python, and statistical significance was evaluated at a threshold of *p* < 0.05.

## 3 Results

### 3.1 Overall Composite Performance (Accuracy) of Model Responses

**FIGURE 1:**
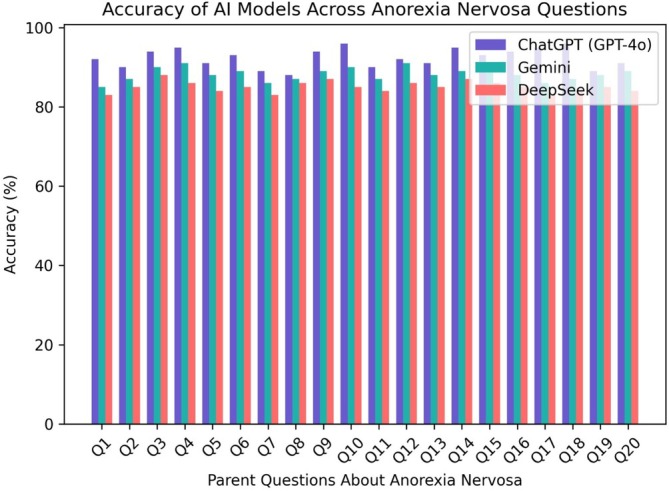
Accuracy of AI models across anorexia nervosa questions. Accuracy scores of ChatGPT (GPT‐4o), Gemini, and DeepSeek for each of the 20 parent‐focused anorexia nervosa questions.

Across the twenty AN‐related questions, all three LLMs demonstrated relatively high levels of overall performance as reflected by the composite accuracy metric. ChatGPT achieved the highest overall accuracy, with a mean score of approximately 92%, followed by Gemini with approximately 88% and DeepSeek with approximately 86%. Accuracy levels varied slightly across individual questions, as illustrated in Figure 1, which presents the accuracy rates for each question (Q1–Q20) across the three models. While the majority of responses were generally consistent with established clinical knowledge, variability was observed particularly in questions related to treatment decisions and long‐term prognosis.

### 3.2 Inter‐Rater Reliability

The scoring process demonstrated high internal consistency, with an ICC of 0.88 (95% CI [0.82, 0.92]), reflecting excellent agreement between the evaluators.

### 3.3 Reproducibility of Responses

**FIGURE 2:**
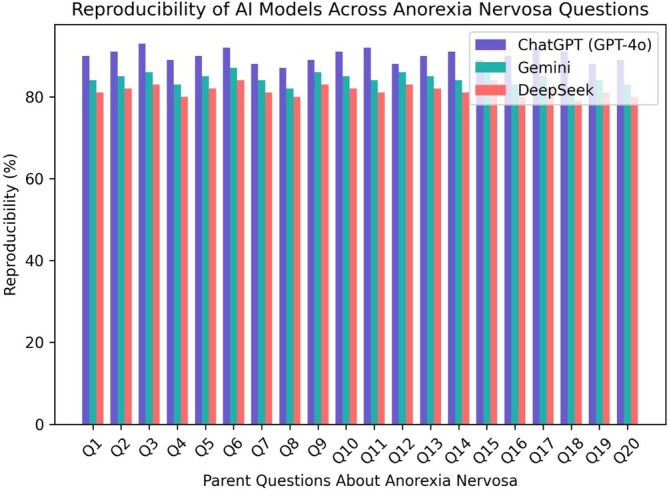
Reproducibility of AI models across anorexia nervosa questions. Reproducibility scores of ChatGPT (GPT‐4o), Gemini, and DeepSeek for each evaluated parent question.

Reproducibility analysis revealed moderate differences between the evaluated models. ChatGPT demonstrated the highest reproducibility rate, with an average score of approximately 90%. Gemini showed a reproducibility rate of approximately 85%, while DeepSeek demonstrated a slightly lower rate of approximately 83%. Although the overall informational content remained largely consistent across repeated prompts, minor differences were observed in the phrasing and emphasis of clinical recommendations. Question‐level reproducibility comparisons across models are illustrated in Figure 2.

### 3.4 Mean Model Performance

**FIGURE 3:**
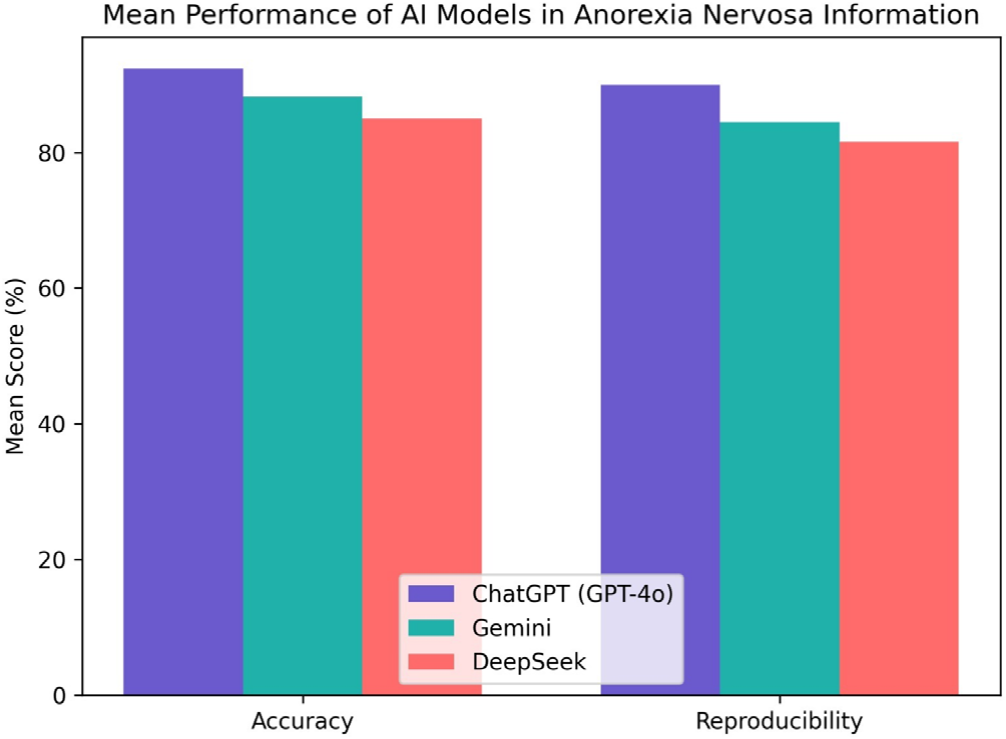
Mean performance of AI models in anorexia nervosa information. Mean accuracy and reproducibility scores of ChatGPT (GPT‐4o), Gemini, and DeepSeek across all evaluated questions.

When accuracy and reproducibility metrics were considered together, ChatGPT demonstrated the highest overall mean performance among the evaluated models. Gemini ranked second, while DeepSeek showed slightly lower overall scores. The mean performance values for each model are presented in Table 3, while the comparative visualization of these mean scores is shown in Figure 3.

**TABLE 3:** Comparison of mean quality, usefulness, and reliability scores across the three large language models using one‐way ANOVA.

Metric	ChatGPT (Mean ± SD)	Gemini (Mean ± SD)	DeepSeek (Mean ± SD)	*p*
Reliability	6.25 ± 0.71	6.40 ± 0.82	5.95 ± 0.94	*p* > 0.05
Quality	4.85 ± 0.36	4.55 ± 0.51	4.55 ± 0.51	*P* > 0.05
Usefulness	6.40 ± 0.75	6.10 ± 0.85	6.05 ± 0.82	*P* > 0.05

*Note:* Values are presented as mean ± standard deviation (SD). Comparisons between models were performed using one‐way ANOVA. Statistical significance was set at *p* < 0.05.

### 3.5 Domain‐Level Performance

**FIGURE 4:**
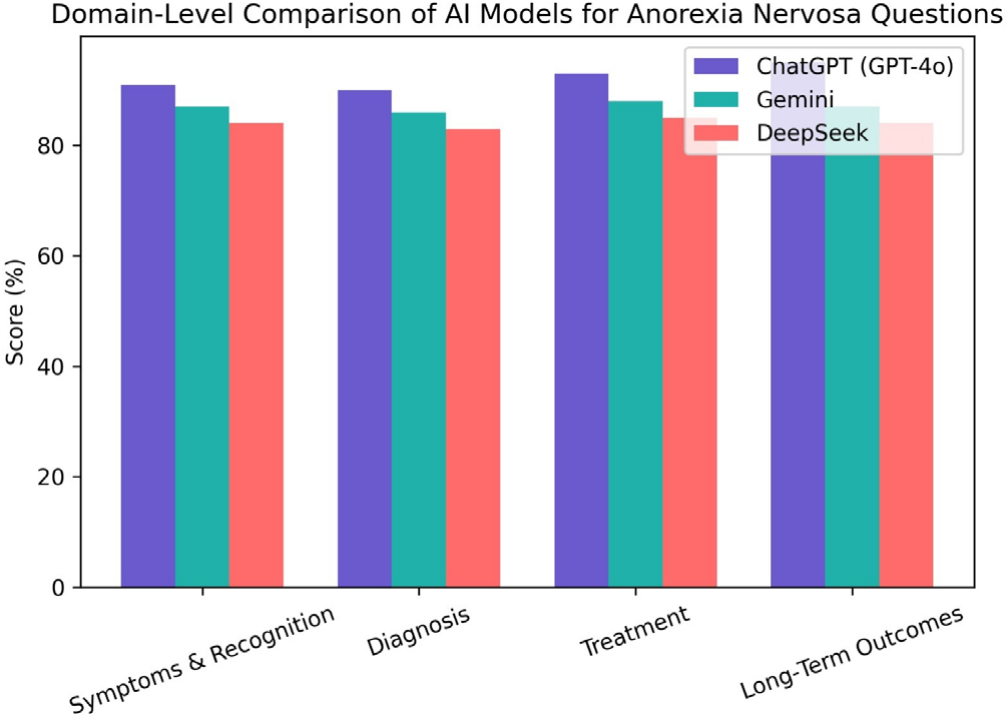
Domain‐level comparison of AI models for anorexia nervosa questions. Performance scores across four domains: Symptoms and recognition, diagnosis, treatment, and long‐term outcomes.

Domain‐level analysis revealed differences in model performance across specific clinical areas. ChatGPT demonstrated the highest scores in the treatment and long‐term outcome domains, indicating stronger alignment with evidence‐based clinical recommendations. Gemini showed relatively consistent performance across diagnostic questions, while DeepSeek demonstrated its relatively strongest performance in the Early Recognition and Symptoms domain, though scores remained lower than those of ChatGPT and Gemini across all four domains. The domain‐level performance scores for each model are illustrated in Figure 4.

### 3.6 Error Analysis

**FIGURE 5:**
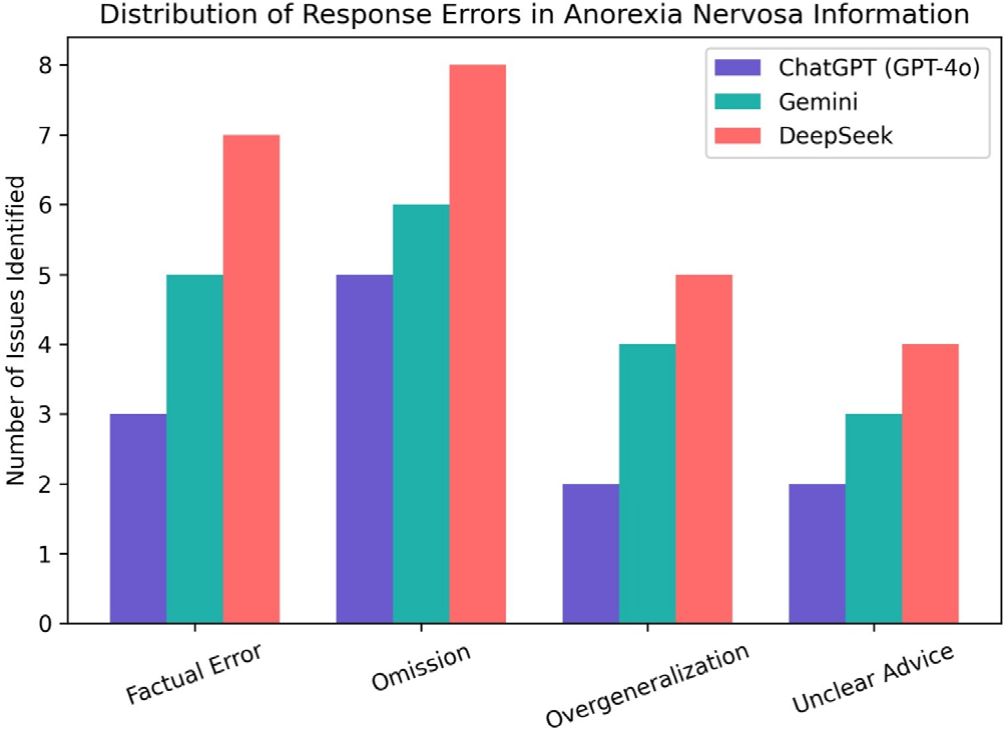
Distribution of response errors in anorexia nervosa information. Number of response errors categorized as factual errors, omissions, overgeneralizations, and unclear advice across the evaluated models.

Qualitative error analysis revealed that the most common limitation across all models was the omission of clinically relevant information, particularly regarding the complexity of multidisciplinary treatment approaches in AN. DeepSeek responses demonstrated the highest frequency of factual inaccuracies, while Gemini responses more frequently included generalized or simplified guidance. ChatGPT responses showed the lowest overall error rate, although occasional simplifications of complex clinical information were observed. The distribution of identified error types across the three models is visually represented in Figure 5.

## 4 Discussion

This study evaluated the accuracy and reproducibility of responses generated by ChatGPT, Google Gemini, and DeepSeek to questions commonly asked by parents of adolescents with AN. All three models demonstrated generally high levels of informational accuracy, with ChatGPT tending to show higher observed performance than the other two models, a pattern consistent with findings from comparable evaluations in parent‐facing pediatric psychiatric contexts (Almulla and Khasawneh 2025; Demir et al. 2025). The overall performance levels observed are numerically higher than those reported in multiple choice questions‐based assessments of child and adolescent psychiatry knowledge (Neubauer et al. 2025), this difference should be interpreted with caution and may likely reflect the structured and well‐defined diagnostic framework of AN rather than a genuine superiority of these models in open‐ended clinical reasoning.

The overall accuracy levels observed in the present study are broadly consistent with—and in some respects numerically higher than—previously reported LLM performance in child and adolescent psychiatry. Neubauer et al. (2025) reported accuracy rates of 68.3% to 78.9% on standardized multiple‐choice questions (MCQ) in child and adolescent psychiatry, noting that feeding and eating disorders were one of the higher‐performing topic areas, and attributed to the relative clarity of diagnostic criteria in this domain compared to other conditions with greater symptom overlap. The relatively high accuracy observed in the present study may reflect the well‐defined diagnostic structure of AN, which appears more agreeable to LLM processing than domains requiring complex clinical reasoning (Neubauer et al. 2025). However, direct comparison between studies is limited by fundamental methodological differences—MCQ and open‐ended parent queries constitute distinct cognitive demands for language models.

Reproducibility analysis indicated that core clinical information remained largely stable across repeated prompts for all three models; however, question‐level data revealed some variability, particularly in the latter half of the question set (Q15–Q20), corresponding to the Long‐Term Outcomes and Family Support domain. A relative decline in model performance was observed across these questions, most pronounced for DeepSeek and Gemini and more evident in reproducibility than in accuracy. This finding is clinically relevant, as the questions within this domain address issues such as recovery prognosis, long‐term consequences, and parental coping strategies, topics for which caregivers are particularly likely to seek information from digital sources between clinical appointments. In conditions such as AN, where the precise characterization of prognosis and recovery can shape family expectations and treatment adherence, even minor inconsistencies in emphasis across sessions may potentially have downstream implications. The relatively lower reproducibility observed for DeepSeek across most questions may warrant attention, as parents consulting this model on different occasions may receive substantively different framings of the same clinical content. One possible explanation is that open‐ended, prognosis‐oriented queries are less effectively supported by the structured diagnostic content that constitutes a large portion of LLM training data. This interpretation is consistent with previous findings in child and adolescent psychiatry indicating weaker model performance on questions requiring more nuanced prognostic reasoning (Neubauer et al. 2025).

Domain‐level analysis revealed variation in model performance across different clinical areas. ChatGPT had the highest observed mean scores in the Long‐Term Outcomes and Family Support domain, whereas Gemini and DeepSeek performed most strongly in the Treatment and Clinical Management domain. Although the magnitude of these differences was modest, they may still be meaningful from an informational perspective, as these topics represent key areas in which families commonly seek guidance during the early stages of AN (Coelho et al. 2021; Oketah et al. 2023). In this context, the generally high level of accuracy observed across models in domains related to treatment and long‐term outcomes may be considered reassuring. Nevertheless, the observed variation between models suggests that the quality and emphasis of information provided to families may still differ depending on the system consulted. Importantly, the Diagnosis and Clinical Assessment domain yielded the lowest accuracy scores across all three models. This finding is particularly relevant, as questions within this domain are closely linked to parental help‐seeking decisions. For parents seeking information independently, imprecise guidance regarding which symptoms warrant professional evaluation or how AN can be distinguished from other conditions associated with weight loss may contribute to delays in accessing appropriate care. Such delays are clinically significant, as delayed diagnosis in AN has been associated with greater illness severity at presentation and less favorable long‐term outcomes (Hebebrand et al. 2024). Therefore, the relatively lower performance observed in the domain most directly related to early identification represents an important limitation that may not be fully captured by descriptive accuracy metrics alone.

The drop in model performance for Long‐Term Outcomes and Family Support likely happens because these topics are less “by the book.” While things like medical diagnoses and treatments follow strict rules found in textbooks, advice on parenting, school support, and emotional recovery is often personal and depends on the situation. Because these topics aren't as clearly documented in the data used to train AI, the models are less reliable when answering questions that require emotional sensitivity and a focus on the family's unique needs. ChatGPT may have shown higher observed performance than Gemini and DeepSeek because of factors such as larger training data, more specific medical tuning, and more human feedback. DeepSeek's higher error rate might be because its training data includes less English clinical content. Meanwhile, Gemini's tendency to give very general answers suggests it was designed for broad accessibility rather than specialized clinical use. These are just theories, however, as this study did not directly analyze the internal tech or training methods of the models.

Error analysis revealed distinct failure profiles across the three models that carry differential clinical implications. Omission of clinically relevant information was the most prevalent error type overall. This finding is consistent with prior observations that LLMs frequently lack depth in developmental and clinical context when addressing parenting‐related health queries (Gugnani et al. 2024; Kim et al. 2025). In the specific context of AN, omissions carry heightened clinical risk: the multidisciplinary nature of treatment, the critical importance of medical monitoring during refeeding, and the central role of family‐based therapy are evidence‐based components that, if underemphasized, could contribute to delayed or incomplete care‐seeking. This risk is compounded by evidence that caregiver burden in AN, particularly dimensions of guilt, social isolation, and nutrition‐related distress, is a meaningful predictor of treatment engagement and weight restoration outcomes in family‐based treatment (Dennhag et al. 2021; Matthews et al. 2023). Responses that omit guidance on caregiver emotional support or fail to emphasize the family's active role in recovery may therefore have consequences beyond misinformation, potentially undermining parental self‐efficacy at a critical juncture. DeepSeek also showed a relatively higher frequency of factual inaccuracies compared to the other models (*n* = 7), while Gemini more frequently produced overgeneralized guidance (*n* = 4) alongside a relatively high omission count. ChatGPT showed the lowest total error burden across all categories, though omissions (*n* = 5) and occasional overgeneralizations (*n* = 2) were still present. These distinct error profiles suggest that the three models differ not only in overall accuracy but in the nature of their failure modes—a distinction with direct practical implications for how clinicians should contextualize each model's output when advising families. It should also be noted that LLMs have been shown to generate fabricated or inaccurate citations at non‐trivial rates, particularly for mental health topics with lower scientific visibility (Linardon et al. 2025). Although the present study evaluated informational accuracy rather than citation integrity, this broader limitation of LLM outputs warrants consideration when interpreting the reliability of AI‐generated health content.

Taken together, the findings of this study suggest that LLMs, and ChatGPT in particular, can provide parents with generally accurate foundational information about AN. However, the consistent presence of omissions across all models, combined with condition‐specific risks associated with incomplete guidance in a high‐stakes clinical context, underscores the importance of positioning these tools as complements to, rather than substitutes for, professional clinical consultation. This conclusion aligns with the broader consensus emerging from the LLM mental health literature: while models demonstrate promising informational capabilities, none currently meet the standards required for independent clinical guidance (Hanafi et al. 2024; Shah et al. 2025). Parents and caregivers should be encouraged to verify AI‐generated health information with qualified professionals, and clinicians should proactively discuss the role of digital tools in their patients' families' information‐seeking processes, given the well‐documented tendency of parents to consult online resources independently of clinical contact (Bringer et al. 2025; Kubb and Foran 2020).

Several limitations should be acknowledged when interpreting the findings of this study. First, the question set comprised only twenty items and may not fully capture the breadth of parental concerns related to AN in real‐world contexts; a larger and more diverse set of questions could improve generalizability. Second, accuracy assessments were conducted by two clinicians from the same disciplinary background. The absence of a multidisciplinary evaluation panel, including professionals such as dietitians or family therapists, may have limited the range of perspectives considered during the assessment process. Third, model comparisons relied primarily on descriptive statistics, as the relatively small number of questions within each domain precluded formal inferential testing. The application of one‐way ANOVA across only 20 questions may have been insufficient to achieve adequate statistical power, which is consistent with the uniformly non‐significant *p* values observed across all evaluation dimensions. Furthermore, since the same questions were rated across all three models, the assumption of independence underlying one‐way ANOVA may not be fully met; future studies should consider mixed‐effects or repeated measures modeling to better account for the nested structure of such data. Therefore, observed differences between models should be interpreted with caution. Fourth, a further limitation concerns the absence of a standardized written scoring framework for the evaluation dimensions. Although anchor descriptions were provided verbally and scale definitions were outlined prior to the assessment process, the lack of a formal rubric may have introduced interpretive variability between raters in how criteria such as clarity, completeness, and clinical consistency were applied to individual responses. This is particularly relevant for distinguishing between adjacent scale points, where subjective judgment necessarily plays a role. As a consequence, the precision of model comparisons, especially where score differences were modest, should be interpreted with caution. Future studies would benefit from the development and pilot‐testing of structured scoring rubrics, or the adoption of validated quality assessment tools adapted for AI‐generated health content, to improve the objectivity and replicability of response evaluation. Fifth, all prompts were submitted in English, which may limit the ecological validity and generalizability of the findings for Turkish‐speaking caregivers who would predominantly seek health information in their native language. LLM performance is known to vary across languages, and future studies should evaluate model responses in the language of the target population. Sixth, the present study focused on the informational accuracy of AI‐generated responses and did not examine how parents interpret or act upon such information in real‐life decision‐making contexts. Finally, LLMs are continuously updated, and the findings represent a time‐specific evaluation that may not necessarily reflect the performance of future model versions.

A key strength of the present study is the systematic evaluation of multiple widely used LLMs using clinically relevant parent‐centered questions. This approach enabled comparison of accuracy, reproducibility, and error profiles across models within a high‐risk pediatric mental health context. Future research could further expand on these findings in several ways. Studies including a broader range of questions and different prompt structures may provide a more comprehensive understanding of model performance. In addition, the use of expert panels or structured evaluation frameworks may strengthen the assessment of response quality. It would also be valuable to investigate how parents interpret and use AI‐generated information in everyday decision‐making, particularly in relation to seeking professional care.

## 5 Conclusion

LLMs demonstrate promising potential as accessible sources of preliminary health information for parents seeking guidance about AN. In this comparative evaluation, ChatGPT, Gemini, and DeepSeek all provided generally accurate responses to common parent questions, with ChatGPT showing the highest observed performance and consistency within this dataset across accuracy, reproducibility, and error profiles. Nevertheless, the persistent presence of clinically relevant omissions and variability across domains highlights important limitations that may not be fully captured by overall accuracy metrics. In a disorder such as AN, where early recognition, timely intervention, and sustained family engagement are critical, partial or inconsistently framed information may potentially influence parental understanding and help‐seeking behavior. Accordingly, while LLMs may serve as useful supplementary informational tools, they should not be considered substitutes for professional clinical guidance. Clinicians should remain aware that families may increasingly consult AI‐generated resources when navigating eating disorder concerns and should proactively support caregivers in interpreting such information within an evidence‐based clinical framework. Further research examining real‐world caregiver interactions with AI systems, as well as strategies to improve the clinical completeness and safety of model responses, will be important for determining the appropriate role of these technologies in pediatric eating disorder education and support.

## Author Contributions

**Serkan Turan:** conceptualization, supervision, project administration, writing – review and editing. **Caner Mutlu:** methodology, visualization. **Esra Rabia Taşpolat:** resources, writing – original draft, writing – review and editing. **Celal Yeşilkaya:** conceptualization, methodology, investigation, formal analysis, writing – original draft, data curation. **Hande Kırışman Keleş:** validation, writing – review and editing, writing – original draft.

## Lived Experience Involvement Statement

No specific efforts were undertaken to involve persons with lived experience in the study design or execution, or in the preparation of this manuscript.

## Funding

The authors have nothing to report.

## Disclosure

No previously published material requiring permission for reproduction was used in this manuscript.

## Ethics Statement

The study involved only the evaluation of AI‐generated responses and did not include human participants, patient data, or identifiable information; ethical approval was not required.

## Conflicts of Interest

The authors declare no conflicts of interest.

## Data Availability

The data generated and analyzed during this study consist of model‐generated responses to a predefined set of questions. The anonymized response dataset is available from the corresponding author upon reasonable request.
